# Unexpected Prolonged Apnea Following Succinylcholine in a Medically Complex Patient: A Case Report

**DOI:** 10.7759/cureus.114907

**Published:** 2026-08-21

**Authors:** Sharlette Lynch

**Affiliations:** 1 Anesthesiology, Overlook Medical Center, Summit, USA

**Keywords:** butyrylcholinesterase deficiency, delayed emergence, prolonged apnea, pseudocholinesterase deficiency, qualitative train-of-four, succinylcholine

## Abstract

Prolonged apnea after succinylcholine should prompt consideration of impaired drug metabolism, including reduced butyrylcholinesterase activity, while other causes of delayed emergence are evaluated. We report a 69-year-old, 72.7-kg man with severe systemic illness and hypoalbuminemia who underwent esophagogastroduodenoscopy and colonoscopy under total intravenous general anesthesia. Induction included propofol, fentanyl, and succinylcholine 100 mg. Approximately two hours after the initial succinylcholine dose, the patient was extubated but developed profound ventilatory weakness, with a respiratory rate of approximately three breaths per minute, tidal volumes of approximately 30 mL, and inability to sustain a head lift. Four qualitative train-of-four responses were present without subjectively detectable fade; no quantitative ratio was available. Naloxone improved alertness but did not restore adequate ventilation. Emergency reintubation was performed with an additional 80 mg of succinylcholine. Head computed tomography demonstrated no acute intracranial abnormality, and the patient was transferred to the intensive care unit for supportive ventilation. Respiratory strength returned gradually, and definitive extubation occurred at 21:25, approximately five hours and 37 minutes after reintubation. The profound weakness documented before the second succinylcholine dose raised concern for a prolonged effect of the initial dose. Severe hypoalbuminemia and systemic illness supported possible acquired reduction in butyrylcholinesterase activity, although inherited deficiency could not be excluded. Because enzyme activity, dibucaine inhibition, genetic testing, and quantitative neuromuscular monitoring were not obtained, the diagnosis remained presumptive. This case emphasizes that four qualitative twitches do not establish adequate neuromuscular recovery and that persistent ventilatory failure after improved consciousness warrants continued evaluation for neuromuscular blockade.

## Introduction

Succinylcholine is a depolarizing neuromuscular blocking agent used when rapid-onset, short-duration paralysis is desired. Its normally brief clinical effect depends primarily on hydrolysis by plasma butyrylcholinesterase, also known as pseudocholinesterase. Markedly reduced enzyme activity can prolong paralysis from minutes to hours and necessitate postoperative mechanical ventilation [1-2]. Deficiency may be inherited through butyrylcholinesterase (BCHE) variants or acquired in association with liver dysfunction, malnutrition, malignancy, systemic inflammation, renal disease, burns, pregnancy, and selected medications [1-3].

Delayed emergence is frequently multifactorial. Residual opioid or hypnotic effect, hypercapnia, metabolic abnormalities, neurologic injury, and residual neuromuscular blockade may coexist. Qualitative train-of-four assessment cannot confirm full recovery because four visible or palpable twitches may be present despite clinically important weakness. Quantitative monitoring with confirmation of a train-of-four ratio of at least 0.9 at the adductor pollicis is recommended before extubation [4]. We describe severe postoperative ventilatory weakness after succinylcholine in a medically complex patient with severe hypoalbuminemia, emphasizing the diagnostic limitations of qualitative monitoring and the need to distinguish improved consciousness from recovery of respiratory muscle function.

## Case presentation

A 69-year-old man weighing 72.7 kg, with a body mass index of approximately 23 kg/m², underwent inpatient esophagogastroduodenoscopy and colonoscopy. He was classified as American Society of Anesthesiologists (ASA) Physical Status III. His medical history included asthma, bronchitis, painless jaundice, pancreatitis following endoscopic retrograde cholangiopancreatography (ERCP), a 14-cm peripancreatic fluid collection, ascites, partial small- and large-bowel obstruction, a left pleural effusion after thoracentesis, *Clostridioides difficile* colitis, and evaluation for a possible intra-abdominal malignancy. Prior anesthetics had not produced a known complication; however, the neuromuscular blocking agents used during those anesthetics were unavailable.

Preprocedure vital signs were blood pressure 128/87 mm Hg, heart rate 93 beats per minute, respiratory rate 18 breaths per minute, and oxygen saturation 96% on room air. Relevant laboratory findings included leukocytosis, anemia, mild hyponatremia, mild hypocalcemia, hyperglycemia, mild coagulation abnormalities, and severe hypoalbuminemia. The selected preprocedure laboratory values, clinical interpretations, and institutional reference ranges are presented in Table 1.

**Table 1:** Selected Preprocedure Laboratory Values and Reference Ranges Reference ranges are those reported by the treating institution’s laboratory.

Variable	Value	Clinical interpretation	Reference range
White blood cell count	16.88 × 10⁹/L	Leukocytosis	4.50-11.00 × 10⁹/L
Hemoglobin	11.0 g/dL	Anemia	14.0-17.0 g/dL
Hematocrit	34.9%	Decreased	39.0%-50.0%
Mean corpuscular hemoglobin	26.3 pg	Decreased	27.0-33.0 pg
Platelets	419 × 10⁹/L	Within reference range	150-450 × 10⁹/L
Sodium	134 mmol/L	Mild hyponatremia	135-145 mmol/L
Potassium	3.8 mmol/L	Within reference range	3.2-4.9 mmol/L
Chloride	103 mmol/L	Within reference range	95-110 mmol/L
Bicarbonate	22 mmol/L	Within reference range	21-32 mmol/L
Glucose	134 mg/dL	Elevated	70-100 mg/dL
Blood urea nitrogen	17 mg/dL	Within reference range	8-23 mg/dL
Creatinine	0.64 mg/dL	Within reference range	0.60-1.30 mg/dL
Calcium	8.2 mg/dL	Mild hypocalcemia	8.5-10.1 mg/dL
Phosphorus	2.6 mg/dL	Within reference range	2.5-4.8 mg/dL
Magnesium	2.5 mg/dL	Upper limit of normal	1.5-2.5 mg/dL
Albumin	2.4 g/dL	Severe hypoalbuminemia	3.5-5.2 g/dL
Alkaline phosphatase	133 U/L	Mildly elevated	40-129 U/L
Aspartate aminotransferase	27 U/L	Within reference range	15-37 U/L
Alanine aminotransferase	16 U/L	Within reference range	10-45 U/L
Total bilirubin	0.4 mg/dL	Within reference range	0.0-1.1 mg/dL
Prothrombin time	15.9 seconds	Mildly prolonged	12.0-15.0 seconds
International normalized ratio	1.21	Mildly elevated	0.90-1.20
Activated partial thromboplastin time	26.7 seconds	Within reference range	23.0-37.0 seconds

General anesthesia was induced with propofol, fentanyl 50 µg, and succinylcholine 100 mg (1.38 mg/kg). No benzodiazepine or other sedative medication was administered. The trachea was intubated with an 8.0-mm endotracheal tube using video laryngoscopy, with a grade I view and successful placement on the first attempt. Total intravenous anesthesia was maintained with propofol; the documented cumulative propofol dose was approximately 320 mg. No nondepolarizing neuromuscular blocking agent was documented during the initial anesthetic.

The procedures began at 13:48 and concluded at 14:38. The patient remained intubated after completion of the procedures and underwent initial extubation at 15:38, approximately two hours after the first succinylcholine dose. Immediately after extubation, he developed profound ventilatory weakness rather than apparent upper airway obstruction. His spontaneous respiratory rate was approximately three breaths per minute, generated tidal volumes were approximately 30 mL, and he was unable to sustain a head lift. A peripheral nerve stimulator demonstrated four qualitative train-of-four responses without subjectively appreciable fade. The monitor did not provide a quantitative train-of-four ratio.

**Figure 1:**
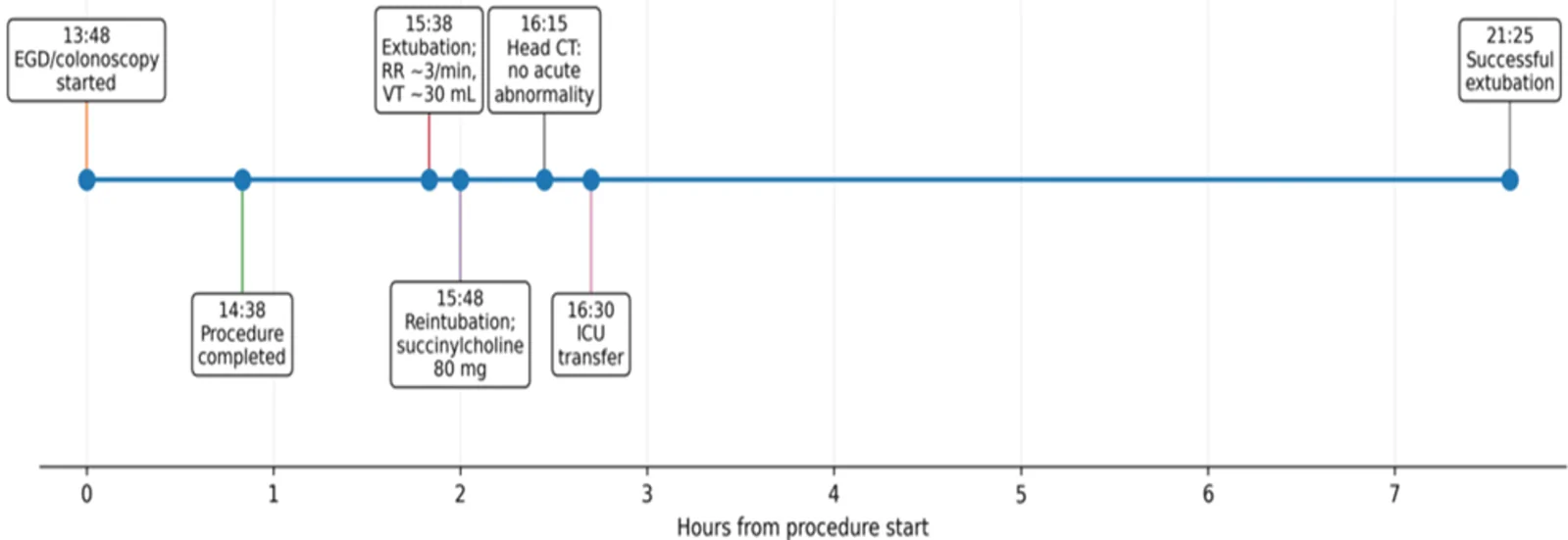
Clinical Course From Procedure Start to Definitive Extubation The timeline summarizes the major procedural, airway, imaging, and intensive care events. EGD, esophagogastroduodenoscopy; RR, respiratory rate; VT, tidal volume; CT, computed tomography; ICU, intensive care unit.

The patient was also poorly responsive. Naloxone was administered in divided doses totaling approximately 0.16 mg, after which alertness improved; however, adequate spontaneous ventilation did not return. Emergency reintubation was performed at approximately 15:48 using an additional 80 mg of succinylcholine (1.10 mg/kg), resulting in a cumulative dose of 180 mg (2.48 mg/kg). He was transferred intubated to the intensive care unit at approximately 16:30 for supportive mechanical ventilation. Spontaneous respiratory strength gradually returned, and definitive extubation occurred at 21:25, approximately five hours and 37 minutes after reintubation. He was subsequently documented as awake, alert, ambulatory, and breathing room air without persistent neurologic deficit. The chronological sequence of procedural, airway, imaging, and intensive care events is summarized in Figure 1.

**Figure 2:**
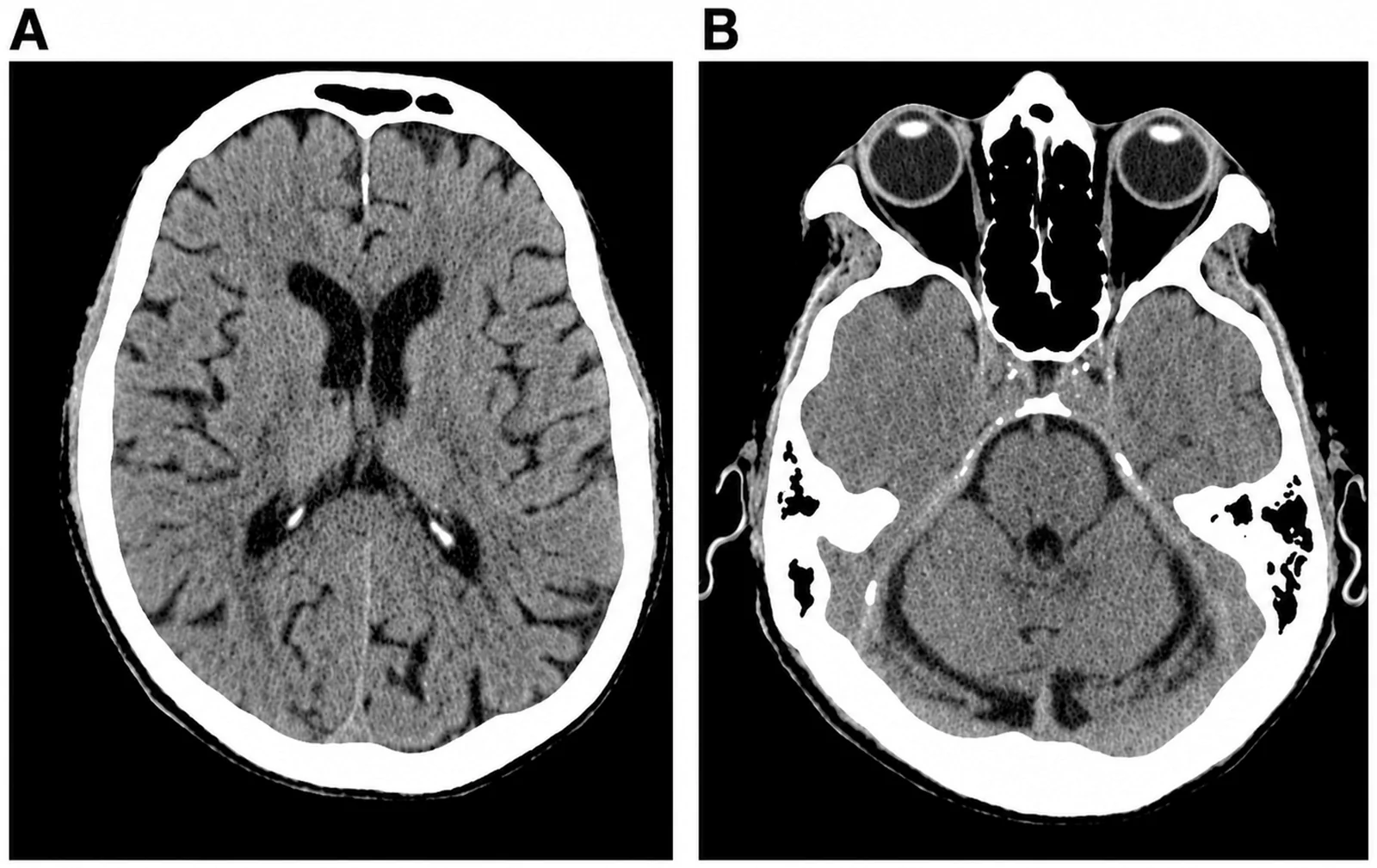
Representative Noncontrast Head Computed Tomography Images Axial noncontrast head computed tomography images obtained after emergency reintubation. (A) Axial image at the level of the lateral ventricles demonstrates preserved ventricular symmetry without acute intracranial hemorrhage, significant mass effect, or midline shift. (B) Axial image through the posterior fossa demonstrates no acute intracranial abnormality. Mild nonspecific white matter changes were reported on the complete examination.

Because the patient remained poorly responsive with persistent ventilatory failure after naloxone administration, an urgent noncontrast computed tomography scan of the head was obtained at approximately 16:15 to evaluate for an acute central neurologic cause of delayed recovery. The examination demonstrated no acute intracranial hemorrhage, significant mass effect, or midline shift. Ventricular size and sulcal appearance were within normal limits, and gray-white matter differentiation was grossly preserved. Mild nonspecific periventricular white matter changes were present, but no acute structural intracranial abnormality was identified to explain the postoperative respiratory failure. Representative axial images are shown in Figure 2.

The profound ventilatory weakness observed before emergency reintubation raised concern for impaired succinylcholine metabolism. Serum butyrylcholinesterase activity, dibucaine inhibition testing, fluoride inhibition testing, and BCHE genetic analysis were not obtained. Accordingly, neither inherited nor acquired butyrylcholinesterase deficiency could be confirmed.

## Discussion

This case demonstrates an important clinical distinction: improved consciousness after opioid antagonism did not produce recovery of adequate ventilation. Residual fentanyl likely contributed to depressed responsiveness, but it did not adequately explain the continued inability to sustain a head lift, the extremely low tidal volumes, or the prolonged requirement for mechanical ventilation. Residual propofol effect and reduced respiratory reserve related to pleural, abdominal, infectious, and systemic illness may also have contributed. Nevertheless, profound weakness was documented approximately 2 hours after the initial succinylcholine dose and before administration of the second dose, making prolonged neuromuscular effect of the initial dose a principal diagnostic consideration.

Four qualitative train-of-four responses without subjectively detectable fade did not exclude clinically important weakness. Visual and tactile assessment cannot reliably distinguish complete recovery from residual block at higher train-of-four ratios, and recovery should be confirmed quantitatively at the adductor pollicis with a ratio of at least 0.9 [4]. In this patient, a respiratory rate of approximately 3 breaths per minute, tidal volumes of approximately 30 mL, and inability to sustain a head lift were incompatible with adequate clinical recovery despite a qualitative count of four. The absence of visible fade may also be observed during phase I depolarizing block and therefore was not reassuring in the context of severe clinical weakness.

Severe hypoalbuminemia was interpreted as a marker of systemic disease and impaired protein nutritional status rather than as proof of the mechanism. Butyrylcholinesterase is synthesized in the liver, and reduced activity has been described with malnutrition, malignancy, chronic infection, systemic inflammation, and impaired hepatic protein synthesis [1-3]. Normal aminotransferases and bilirubin made active severe hepatocellular injury less evident but did not exclude reduced enzyme production or activity. An inherited BCHE variant also remained possible because prior uncomplicated anesthesia does not establish previous exposure to succinylcholine or mivacurium.

The additional 80-mg succinylcholine dose administered for emergency reintubation was a major confounder and may have extended the subsequent period of weakness if metabolism was already impaired. However, the objective respiratory failure that necessitated reintubation occurred before that second dose. When prolonged succinylcholine effect is suspected, additional succinylcholine should be avoided when clinically feasible. Management is supportive and includes airway protection, mechanical ventilation, and adequate sedation and amnesia until neuromuscular function returns [1,2].

Definitive evaluation includes measurement of plasma butyrylcholinesterase activity and phenotypic inhibition testing, most commonly the dibucaine number. Reduced enzyme activity identifies low circulating function but does not by itself distinguish acquired from inherited deficiency. The dibucaine number can identify atypical phenotypes, and BCHE genetic testing may be considered when hereditary deficiency is suspected [1-3,5]. The absence of these tests is the principal limitation of this report. Additional limitations include the lack of quantitative neuromuscular monitoring and unavailable arterial blood gas and formal respiratory mechanics at the failed extubation.

Published reports describe prolonged postoperative ventilation after succinylcholine in patients subsequently found to have reduced butyrylcholinesterase activity [5-7]. The present case is educational because delayed emergence appeared mixed: naloxone improved alertness, whereas profound ventilatory weakness persisted. This distinction may help clinicians avoid attributing all postoperative hypoventilation to opioids when neuromuscular dysfunction remains possible.

## Conclusions

Unexpected apnea after succinylcholine should prompt consideration of persistent neuromuscular blockade even when opioid or hypnotic effects may coexist. Four qualitative train-of-four twitches do not establish complete recovery, and quantitative confirmation of a ratio of at least 0.9 is preferred before extubation. In this patient, objective respiratory weakness was present before the second succinylcholine dose and persisted despite improved alertness after naloxone, supporting a suspected prolonged succinylcholine effect. Severe hypoalbuminemia and systemic illness made acquired reduction in butyrylcholinesterase activity biologically plausible, but confirmatory biochemical and genetic testing was not obtained; therefore, pseudocholinesterase deficiency cannot be stated as a definitive diagnosis. The event should be documented prominently, and the patient should be counseled to avoid succinylcholine and mivacurium until formal evaluation with butyrylcholinesterase activity and a dibucaine number is completed.
